# A Protocol for a Scoping Review Comparative Bibliometric Analysis of Infectious Disease Research in Africa

**DOI:** 10.3389/fpubh.2021.802428

**Published:** 2022-01-03

**Authors:** Adrianna Perryman, Gebremedhin Beedemariam Gebretekle, Adeteju Ogunbameru, Joanna M. Bielecki, Beate Sander

**Affiliations:** ^1^School of Global Health, York University, Toronto, ON, Canada; ^2^Toronto Health Economics and Technology Assessment (THETA) Collaborative, Toronto General Hospital Research Institute (TGHRI), University Health Network, Toronto, ON, Canada; ^3^University Health Network (UHN), Toronto, ON, Canada; ^4^Institute of Health Policy, Management and Evaluation (IHPME), University of Toronto, Toronto, ON, Canada; ^5^Program for Health System and Technology Evaluation, Toronto General Hospital Research Institute (TGHRI), University Health Network (UHN), Toronto, ON, Canada; ^6^Public Health Ontario, Toronto, ON, Canada; ^7^ICES, Toronto, ON, Canada; ^8^Population Health Economics Research (PHER) Program, Toronto, ON, Canada

**Keywords:** COVID-19, bibliometric, Zika (ZIKV), Ebola (EBOV), influenza, tuberculosis, infectious disease, Africa

## Abstract

**Introduction:** Evidence on authorship trends of health research conducted about or in Africa shows that there is a lack of local researchers in the first and last authorship positions, with high income country collaborations taking up these positions. The differences in authorship calls into question power imbalances in global health research and who benefits from the production of new discoveries and innovations. Health studies may further go on to inform policy and clinical practice within the region having an impact on public health. This paper aims to compare the differences in authorship between COVID-19 and relevant infectious diseases in Africa.

**Materials and Methods:** We will conduct a bibliometric analysis comparing authorship for COVID-19 research during a public health emergency with authorship for four other infectious diseases of relevance to Africa namely: Ebola, Zika Virus (ZIKV), Tuberculosis (TB) and Influenza. Our scoping review will follow the framework developed by Arksey and O'Malley and reviewed by Levac et al. We will search MEDLINE (Ovid), African Index Medicus (AIM), Eastern Mediterranean Region (IMEMR) Index Medicus, Embase (Ovid), and Web of Science (Clarivate). We will compare the different trends of disease research between the selected diseases. This study is registered with OSF registries and is licensed with the Academic Free License version 3.0. The open science registration number is 10.17605/OSF.IO/5ZPGN.

## Introduction

Infectious diseases are a global health concern; however, there exist grave disease disparities between the Global North and Global South. According to the World Health Organization, communicable diseases, maternal causes, and nutritional deficiencies accounted for ~53% of all deaths in Africa in 2019, in comparison to 10% in the Americas and 5% in Europe (1). 25% of the global burden of disease is in Africa, despite making up 15% of the world's population (2). The burden of preventable infectious diseases such as HIV/AIDS, malaria, and TB remain high in the region (1, 3) with increased morbidity, mortality, and economic inequality due to health care cost (3). Context-specific research on the burden of disease and interventions is imperative to addressing these disparities and improving health outcomes. The number of publications from the African region are increasing; however, there still exist gaps in regional research outputs. African researchers face many systemic barriers, which limits the production of research and results in the need for partnership and collaborations among Africa and collaborators from low-income (LIC) and high-income countries (HIC). Barriers include limited high degree scholars that can supervise junior researchers, barriers to accessing infrastructure to carry out specific research tasks, weak governance, and insufficient funds to conducting research (4).

Partnerships and collaborations between Africa and HIC are beneficial and have the capacity to improve global health research and solve health challenges (5). Research, however, points to an unequal distribution of first/last authorship positions held by researchers in HICs compared to authors from the country of research origin (6). Differences in leadership roles demonstrate how global health research may be perpetuating colonial practices (6), creating gaps in who is rewarded and is able to benefit from the research being conducted. Authorship holds important value in the scientific community, allowing individual career advancement, additional funding and leadership opportunities (6). Research that is led by local researchers can also inform policy and help create health solutions that are relevant to the cultural and political context of the local population (2). Correspondingly, a study reported that <10% of the global investment in health research is spent in LMICs despite these regions experiencing 90% of preventable mortality (7). As funding remains a barrier to health research in these regions, inequalities may persist. Subsequently, when funding is sourced from HICs, this may allow researchers from these regions to lead research goals and outputs (7). The lack of investment and leadership opportunities results in regions such as those in Africa to be unable to lead many publications further contributing to the systemic barriers that authors face in research production.

A systematic review of authorship trends for infectious disease research conducted in Africa found that 80% of articles published in infectious disease journals worldwide had authors from the USA and Western Europe (5). This study also reported that only 49.7% of selected articles had a first author from an African Institution and 41.3% had a last author affiliated with an African Institution (5). Another study found that one in eight publications coauthored from the USA, Canada or Europe had no authors from the country of focus (7). In addition, a study on dominance and leadership trends in research activities noted that authors classified to be affiliated with institutions in low/medium human development nations (particularly low-income or middle-income countries) were listed as first authors on only 40–53% of publications in the topic of infectious disease whereas high/very high human development nations (United States, Canada, UK, Germany, France, Chine and Brazil) led 78–89% of papers in this field (4). Similarly, 76–90% of authors from high/very high human developed institutions were listed in the last authorship position whereas, 36–50% for low/medium development countries (4).

A bibliometric analysis on COVID-19 research in Africa found that 90.3% of articles published had at least one African researcher, 78.5% of the papers had an African researcher in the first author position and 63.5% in the last author position (8), higher than authorship trends for other infectious disease research (5–7). To further explore African research productivity, we will be conducting a bibliometric analysis on infectious disease research about or in Africa. We have selected four infectious diseases [Ebola, Influenza, Tuberculosis (TB) and Zika Virus (ZIKV)] to be compared to COVID-19 to further understand these trends of health research in Africa.

By comparing authorship trends, we aim to contribute to the growing literature on potential power imbalances in health research in Africa, and to inform equitable global health research collaborations between researchers from high-income countries and researchers from Africa. Our analysis will also highlight gaps in infectious disease research locality to encourage a broader scope in disease research which will ultimately broaden our understanding of the epidemiology of infectious diseases and inform interventions.

## Materials and Methods

The scoping review will be guided by the framework proposed by Arksey and O'Malley, which has been refined by Levac et al. and Joanna Briggs Institute (9, 10). This study will follow the scoping review process except for stage 6, the consultation exercise, which will be excluded due to resource and time constraints. We will be conducting the bibliometric analysis using the VOSViewer (11) and Bibliometrixs R-program (12). VOSviewer will be used to conduct co-authorship maps. Due to some of the limitations of the VOSViewer software we will be using BibliometrixR to gather information on most prolific countries, authors, and institutions for each of the infectious diseases.

### Stage 1: Identifying the Research Question

The purpose of this study is to understand the landscape of first and last authorship patterns of health research in Africa. We will be comparing the authorship trends of five infectious diseases: Ebola, Influenza, TB and ZIKV to COVID-19. TB and Influenza were chosen as comparators because of their similarity in disease transmission to COVID-19 (13). TB was also selected to capture endemic diseases that exist in Africa (14). Influenza was also selected along with ZIKV because both pathogens are known to have caused previous pandemics and have a potential of causing pandemics in the future (15, 16). Ebola virus was selected due to being unique to Africa and having substantial outbreaks in this region (16). We will explore the potential gaps in research locality, identify co-author collaborations and institution affiliation trends in infectious disease research.

A systematic search and a review of the literature was conducted to understand the landscape of authorship trends in health research. Based on our pilot search of the literature our overarching research question is “What are the authorship trends of infectious disease research in Africa” and our specific research questions will consider:

1. What are the most productive countries in infectious disease research?
2. What are the most productive organizations in infectious disease research?
3. Who are the most productive authors?
4. What country collaborations have first or last authorship by African researchers?
5. What types of studies are authored, first or last, by African researchers?
6. What types of collaborations receive the most citations?
7. How do authorship trends for Ebola, Influenza, TB and ZIKV compare to authorship trends for COVID-19 health research in Africa?

### Stage 2: Identifying Relevant Studies

A search strategy was developed by an information specialist. Search terms include MeSH terms for the selected infectious diseases and causative pathogens as well as a full list of countries in the continent of Africa. We have selected five databases to retrieve relevant articles: MEDLINE (Ovid), African Index Medicus (AIM), Eastern Mediterranean Region (IMEMR) Index Medicus, Embase (Ovid) and Web of Science (Clarivate).

We will include research published between January 1, 2019 and March 31, 2021, to describe the most recent trends of infectious disease publications and ensure feasibility.

### Stage 3: Study Selection

Identified studies will be imported into Zotero to manage the references, remove duplicates and combine the data to be imported into VOSviewer (11) and BibliometrixsR (12). Titles and abstracts will be reviewed by three reviewers independently. We will include published peer reviewed articles during the selected time frame. Articles of any study design and language will be included. Editorials, commentaries, conference proceedings, abstracts, and gray literature will not be included. Gray literature was excluded due to the time frame constraints of the study and due to the limited capabilities of analyzing references outside of major databases in the listed software's. To account for studies authored by African researchers that may not have been indexed in major bibliometric databases we have included African Index Medicus. We will also exclude animal studies conducted for veterinarian purposes. Each reviewer will decide whether the article will be included based on the eligibility criteria.

### Stage 4: Charting the Data

The data will be tabulated using Microsoft Excel by extracting the following bibliometric data: authors, author affiliation, the location of the study, year of publication, study design, country of publication, study funders, and the number of times the publication was cited.

### Stage 5: Collating, Summarizing, and Reporting the Results

The results will give us insight on the authorship trends of infectious disease research in Africa. We will use PRISMA for scoping reviews (PRISMA-ScR) (9) checklist to guide the reporting of our study's findings. Results will be reported using quantitative and qualitative measures. The report will also present co-authorship maps and network visualization based on geographical location of authors, most relevant journals, most citated authors, and affiliations. The results for each infectious disease will be compared to analyse the changing patterns of disease related research. Bibliometrix R-program (12) will be used for descriptive data analysis such as the most productive countries and authors. VOSviewer (11) will be used to create co-authorship network maps.

## Discussion

The results of the bibliometric analysis will provide insight on the changing patterns of authorship trends for research on infectious disease over the last 3 years, including COVID-19 in Africa. The scoping review will further, identify potential gaps in research production in Africa and describe low-income and high-income country collaborations in the scope of infectious disease and health research. Our study is limited to the time frame considered and the selected infectious diseases due to resource constraints, impacting generalizability of our findings. This study does not include gray literature which may introduce publication bias. Our study is also limited in our understanding of the relationships between the corresponding author and the location of research origin. This is not something that we are able to investigate given the limitations of bibliometric data and the number of citations that will be included in the study, investigating such relationships would not be feasible. However, reporting on first and last authorship can provide important insights, e.g., by showing a potential focus on capacity building in which local researchers may play a more significant role in the development of research gaining first or last authorship. Lastly, inconsistent bibliometric data recording across journals and databases may limit some results and analysis. A significant strength of the study is that it will investigate multiple infectious diseases which is novel and will provide a more comprehensive understanding of infectious disease research in Africa, considering a range of contexts, from endemic diseases to global public health emergencies. Our rigorous search of literature includes several databases including specific African databases to ensure local research is adequately captured. Overall, this study will provide evidence on authorship trends, collaboration patterns, and research being conducted by HIC and LIC researchers in Africa. Overall, this study will inform policies and practices in global health research with the goal of ensuring equitable partnerships and collaborations.

## Conclusion

This protocol presents the methods in which the study will investigate the changing patterns of first and last authorship in Africa. It describes the search strategy, study selection, analysis and reporting of results that will be conducted. The scoping review is unique in that it will investigate several infectious diseases that are of significance in the African region. Similarly, this work will contribute to identifying relevant gaps in local authorship and high-income country collaborations.

## Author Contributions

AP developed the first and subsequent drafts of the protocol. JB developed the search strategy and advised on the methods of the study. All authors contributed to the development of the study protocol, reviewed, revised, and approved the final draft.

## Funding

This work was supported by the Canada Research Chair in Economic of Infectious Diseases held by BS (CRC-950-232429).

## Conflict of Interest

The authors declare that the research was conducted in the absence of any commercial or financial relationships that could be construed as a potential conflict of interest.

## Publisher's Note

All claims expressed in this article are solely those of the authors and do not necessarily represent those of their affiliated organizations, or those of the publisher, the editors and the reviewers. Any product that may be evaluated in this article, or claim that may be made by its manufacturer, is not guaranteed or endorsed by the publisher.

## Abbreviations

COVID-19: Coronavirus Disease 2019
LICs: low-income countries
LMICs: low-middle income countries
MeSH: medical subject headings
TB: tuberculosis
USA: United States of America
ZIKV: Zika Virus.
